# Pediatric intensive care stress ulcer prevention (PIC-UP): a protocol for a pilot randomized trial

**DOI:** 10.1186/s40814-017-0142-y

**Published:** 2017-05-19

**Authors:** Mark Duffett, Karen Choong, Jennifer Foster, Elaine Gilfoyle, Jacques Lacroix, Nikhil Pai, Lehana Thabane, Deborah J Cook, For The Canadian Critical Care Trials Group

**Affiliations:** 1 Departments of Pediatrics and Clinical Epidemiology and Biostatistics, McMaster University, Hamilton, Ontario Canada; 2 Department of Pediatrics, Dalhousie University, Halifax, Nova Scotia Canada; 3 Department of Paediatrics, University of Calgary, Calgary, Alberta Canada; 4 Department of Pediatrics, Université de Montréal, Montreal, Quebec Canada; 5 Department of Pediatrics, McMaster University, Hamilton, Ontario Canada; 6 Department of Clinical Epidemiology and Biostatistics, McMaster University, Hamilton, Ontario Canada

**Keywords:** Pediatric intensive care, Pediatric critical care, Pantoprazole, Gastrointestinal hemorrhage, Ventilator associated pneumonia

## Abstract

**Background:**

Despite sparse pediatric data on effectiveness, the majority of critically ill children receive medications to prevent gastrointestinal (GI) bleeding. Stress ulcer prophylaxis may have unintended consequences—increasing the risk of nosocomial infections—which may be more serious and common than the bleeding which these drugs are prescribed to prevent. Randomized controlled trials (RCTs) in pediatric critical care are exceptionally challenging to complete, thus a rigorous pilot RCT is crucial. The objective of this pilot RCT is to assess the feasibility of a large multicentre RCT of stress ulcer prophylaxis with pantoprazole to prevent upper GI bleeding vs. placebo.

**Methods:**

A multi-centre blinded pilot RCT of 120 children in six Canadian PICUs. Children expected to require mechanical ventilation for more than 48 h will be randomized to receive intravenous pantoprazole 1 mg/kg or identical placebo once daily until they no longer need mechanical ventilation. We have four feasibility outcomes and will consider the trial successful if we achieve:

1. Effective screening: If >80% of eligible patients are approached for consent.
2. Timely enrollment: if >80% of participants receive their first dose of the assigned study drug within 1 day of becoming eligible.
3. Participant accrual: If the average monthly enrolment is two or more participants per centre per month.
4. Protocol adherence: if >90% of doses are administered according to the protocol.

**Discussion:**

There are many uncertainties about the risks and benefits of stress ulcer prophylaxis. In an era of widespread use—where clinicians prescribe prophylaxis to the more severely ill—a large, rigorous RCT is required. A trial to determine if a strategy of withholding stress ulcer prophylaxis is not inferior to a strategy of routine stress ulcer prophylaxis will be challenging. A carefully designed and implemented pilot trial is essential.

**Trial registration:**

ClinicalTrials.gov:NCT02929563 (Registered October 3, 2016).

## Background

Critically ill children are at risk of upper gastrointestinal (GI) bleeding. In observational studies the incidence of important bleeding ranged from 0.4 to 5% [1–5]. This bleeding is associated with more red blood cell transfusions, longer duration of mechanical ventilation and stay in the pediatric intensive care unit (PICU) as well as increased healthcare costs [6]. The use of acid suppression medications to attempt to decrease risk of GI bleeding—most frequently proton-pump inhibitors (PPIs) or histamine-2 receptor antagonists (H_2_RAs)—is common. In a retrospective study of 42 hospitals in the USA, 60% of children admitted to a PICU received acid suppression. In a prospective observational study of 398 children from five PICUs in Brazil, 78% of children received prophylaxis [7].

Despite frequent use of these agents, previously published RCTs are not sufficient to assess the benefits of prophylaxis—any estimate of effect is uncertain. The three trials (340 children) that reported macroscopic or important bleeding did not find a difference between prophylaxis and no prophylaxis (relative risk [RR] 0.71; 95% confidence interval [CI] 0.42 to 1.19, *p* = 0.19) [8–10]. The confidence interval is wide and the strength of inference is low as there were only 21 bleeding events. Using the Grading of Recommendations, Assessment, Development and Evaluations (GRADE) [11] approach, the quality of the evidence for the outcomes of clinically important bleeding and any overt bleeding was very low [12].

Previously published RCTs are also not sufficient to assess the harms of prophylaxis. Suppressing gastric acid secretion reduces a key host defense against pathogenic bacteria. Accumulating data in other populations confirms an increased risks of infection with the use of acid suppression. Of particular concern to critically ill children are ventilator-associated pneumonia (VAP) and *C. difficile* associated disease (CDAD). These serious side effects have not been assessed in critically ill children. The trial reporting VAP was very small and could not exclude an important effect (RR =1.14; 95% CI 0.74 to 1.77, *p* = 0.54) [10]. No trial of stress ulcer prophylaxis has measured CDAD [12].

A large RCT is needed to inform clinicians as they seek to balance the risks and benefits of stress ulcer prophylaxis. Such an RCT will be challenging to conduct. Only 320 RCTs have been published in pediatric critical care to date. They are typically small (median 50 children) and 30% were stopped early: 86% for futility or recruitment [13]. Conducting a pilot trial first is thus a scientifically and ethically responsible approach. The objectives of this pilot trial are to assess the feasibility of a large trial and to evaluate and refine inclusion and exclusion criteria, test study procedures, streamline data collection, and to assess parental and physician acceptability.

## Methods/design

### Study design and setting

**Fig. 1:**
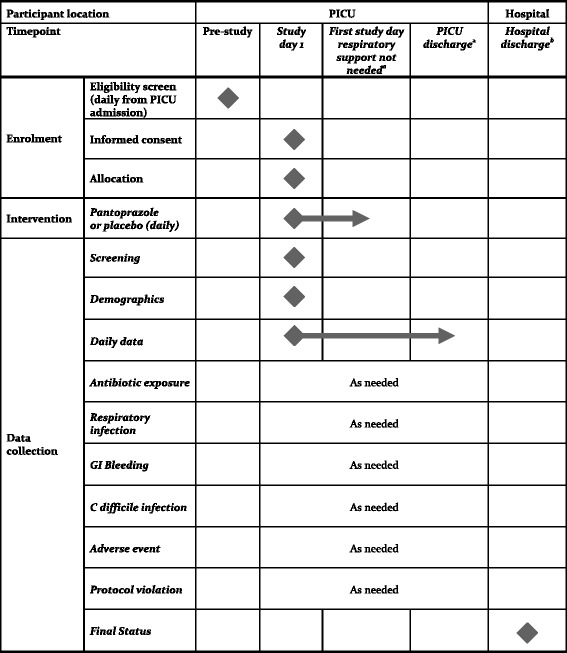
Schedule of enrollment, interventions, and data collection

We are conducting a blinded pilot RCT at six Canadian tertiary centres—anticipating that we can assess feasibility in some the centres that will participate in the large trial and make the results more generalizable beyond a few highly-motivated centres. Figure 1 outlines the schedule of enrolment, interventions, and data collection.

### Study population

We aim to enroll children who will be in the PICU long enough to be at substantial risk of stress ulcer associated bleeding and who will be exposed to the intervention for long enough to accrue any potential benefit or experience any potential harms.

Inclusion criteria:

1. between 12 months and 18 years of age
2. requires respiratory support in the form of invasive mechanical ventilation, non-invasive mechanical ventilation, or high-flow oxygen therapy
3. the attending physician expects the child to require respiratory support for at least 2 more days

We had originally planned to include infants less than 1 year of age as well, but Health Canada did not approve enrolling this age group, citing a paucity of safety data. This will reduce the number of eligible patients and limit the generalizability of the results, as infants are a substantial proportion out population of interest. To avoid delay, we started the trial using 1 year as the minimum age and will re-apply to include infants.

Exclusion criteria:

1. Children who have an indication for acid suppression and should not receive placebo: H_2_RA or PPI use for >1 week in the past month, active GI bleeding, documented severe reflux, active H. pylori infection, severe esophagitis, Zollinger-Ellison syndrome, Barrett’s esophagus, peptic ulcer bleeding within 8 weeks, or are receiving methylprednisolone 15 mg/kg/day or more (or equivalent).
2. are receiving mycophenolate (enteral), methotrexate, nelfinavir, atazanavir, saquinavir, or posaconazole (because of the risk of drug interactions)
3. children with chronic illness with low severity of illness, those receiving chronic ventilation on usual pressure settings and rate, nocturnal or intermittent non-invasive ventilation only, or are eating, nursing, or if chronically fed via feeding tube, are receiving his/her usual feeds
4. received more than 1 daily-dose equivalent of any acid suppressive medication in the PICU
5. are currently enrolled in a potentially confounding trial
6. are known to be pregnant or breastfeeding
7. are known to be allergic to pantoprazole or any other ingredient in the product
8. are not expected to survive this PICU admission because of palliative care or limited life support

### Sample size justification

We will randomize 120 children. We anticipate enrolling over a total of 18 months, with sites starting sequentially over a 6-month period. Factors we considered in estimating the sample size were threefold:

1. Participants per centre: To ensure we are able to assess the feasibility and test study procedures and infrastructure at each site, we will aim to enroll at least 15 participants per centre.
2. Number of centres: To ensure that the results are generalizable beyond a few highly-motivated centres and to reflect the centres that will enroll children in the large trial, six PICUs will recruit participants.
3. Precision of feasibility estimates: To ensure that we will estimate our feasibility measures with sufficient precision, we calculated the number of children required so that the lower bound of the 95% confidence interval was above the threshold of 80% (for both effective screening and timely enrolment, the most important feasibility outcomes). If 105 of 120 participants meet one of those criteria, the lower limit of the 95% confidence interval is 80%.

### Screening

Children may be enrolled in this trial at any point in their PICU stay once they fulfill the eligibility criteria and have none of the exclusion criteria for the first time. Research coordinators will screen all children in the PICU each weekday (and on weekends if possible) and will maintain screening logs including the reasons for exclusion and the reasons why those who were eligible were not approached for consent.

### Consent

Research staff will approach the parents or guardians for consent for their child to participate in this trial. As all children will often be sedated and are critically ill, they will be unable to provide assent. Participants may withdraw from the trial at their request or the request of their parent or guardian. We will include all data collected prior to the withdrawal of consent. The median consent rate in published pediatric critical care RCTs is 90% [14]. We anticipate that more than 80% of parents will provide consent given that we are testing a well-tolerated intervention that is in common use and the focus on preventing adverse effects from medications. We will monitor the consent rate and solicit feedback from parents to better understand the modifiable reasons for consent refusal and develop strategies to address these.

### Allocation

The research pharmacy staff at each site will randomize participants to pantoprazole or placebo in a 1:1 ratio using REDCap (projectredcap.org). The randomization schedule is computer-generated, stratified by centre, uses randomly varying, undisclosed blocks and is concealed from all other study staff and researchers.

### Trial intervention

Participants will be randomized to receive intravenous pantoprazole or identical placebo once daily. The intervention will continue until the participants no longer need mechanical ventilation or any respiratory support—to a maximum of 30 days or until PICU discharge. The study medication will be stopped if the attending physicians believe it is imperative to start open-label acid suppressive medication for GI bleeding or if IV access is lost and there is no longer a clinical need to re-establish access. When the study intervention is discontinued (for any reason), the treating team can follow their usual practice with respect to acid suppression.

In the absence of pediatric-specific data, we have chosen a PPI because this class may be more effective in preventing clinically important bleeding in adults than H_2_RAs (RR 0.36; 95% CI 0.19–0.68; *p* = 0.002) [15]. Pantoprazole is the only intravenous PPI marketed in Canada. Intravenous administration is necessary because oral or enteral administration will not always be feasible and the extent of absorption is uncertain in many critically ill children. Based on data from McMaster Children’s Hospital, we anticipate that participants would receive the study intervention for a median of 5 to 6 days.

Participants will receive 1 mg/kg (maximum 40 mg) of pantoprazole or placebo once daily [16, 17]. Doses will be calculated on the patient’s admission weight and rounded to the nearest mg. Doses will not be adjusted to account for weight changes in the PICU. The placebo will be an equivalent volume of 0.9% saline. The research pharmacist at each site will supply individualized unit-dose syringes of pantoprazole 4 mg/mL (in 0.9% saline) or placebo (an equivalent volume 0.9% saline) daily for each participant. Individual sites may also elect to prepare numbered kits, containing the first dose of the study intervention, which will be stored in the PICU and opened in sequence once each participant is enrolled. This will permit enrollment at any time of day and reduce the time gap between consent and study drug administration. The PICU nursing staff will administer the pantoprazole or placebo intravenously over 1 h or less using their hospital’s usual practice. The first dose will be given as soon as possible after randomization. Subsequent doses will be given once daily at a time of day consistent with the hospital’s usual practice.

### Blinding

Pantoprazole solution is clear and colourless. All clinicians, research staff, and parents and guardians will be unaware of the treatment allocation. Only the Research Pharmacist at each site will be aware of the treatment allocation. The group assignment will not be revealed upon request of the treating team as the treatment for adverse events and accidental overdoses is symptomatic and knowledge of the group assignment will not be clinically useful.

### Outcomes

The pilot trial will focus on four feasibility outcomes. We will consider it to be successful if we achieve:

1. *Effective screening*: If >80% of eligible patients are approached for consent. Children may be eligible but not enrolled if the attending physician declines or if research staff are unavailable or are unable to coordinate meeting with the parents or guardians while the child remains eligible.
2. *Timely enrollment*: if >80% of participants receive their first dose of the assigned study drug within 1 day of becoming eligible.
3. *Participant accrual*: If the average monthly enrolment is 2 or more participants per centre per month.
4. *Protocol adherence*: if >90% of doses are administered according to the protocol.

Although not the focus of the pilot trial, we will collect the following clinical outcomes to test and refine the data collection process for the main trial.

1. *Clinically important bleeding*: Overt bleeding from the GI tract (can be hematemesis, nasogastric blood, melena, hematochezia) associated with one of the following within 24 h: a decrease in hemoglobin of >20 g/L, hypotension (a decrease in systolic blood pressure of >10 mmHg or the need for new or increased doses of vasoactive medications), tachycardia (an increase in heart rate of >20 beats per minute) or a red blood cell transfusion. All bleeding events will be assessed by two blinded adjudicators to determine if they meet these criteria. The definition has been validated, shows excellent inter-rater agreement (kappa = 0.76), and has been used in adult RCTs [18, 19].
2. *VAP*: As assessed by two blinded adjudicators using the Centers for Disease Control Criteria [20]
3. *CDAD:* Diarrhea with a positive test (using each hospital’s usual laboratory methods) for C. difficile.
4. *Other clinical outcomes*: Death in the PICU, endoscopy or surgery for bleeding, transfusions, minor GI bleeding, treatments used for VAP, CDAD, and GI bleeding, PICU and hospital length of stay, and duration of mechanical ventilation.

We will collect daily data for a maximum of 30 days after randomization. After that point, we will only collect the duration of PICU and hospital stay, vital status and incidence of CDAD. Research staff will enter the data directly into a secure database (REDCap) that includes both range checks and logic checks and alerts users to any missing data.

### Participant safety and reporting of adverse effects

Adverse effects with pantoprazole are generally mild and transient. In RCTs conducted outside of the ICU, 1–3% of participants reported GI disorders (constipation, diarrhea, nausea, vomiting, bloating, and discomfort), headache, skin reactions, and injection site reactions [21]. Critically ill patients are at high risk of serious adverse events and the usual approach of reporting all serious adverse events to participating centres’ Research Ethics Boards (REB) would result in large numbers of reports of events not related to the trial intervention, but rather reflect the underlying disease process or expected complications of critical illness [22]. The most likely adverse effects associated with stress ulcer prophylaxis and withholding stress ulcer prophylaxis are bleeding and nosocomial infection, both of which are captured as outcomes and thus not reported as serious adverse events. Only serious adverse events that might reasonably be a consequence of participation in the trial and are judged by the investigators not due to the underlying disease or expected complications of critical illness will be reported to Health Canada, our study’s Data Safety Monitoring Committee (DSMC) and the centre’s REB.

The DSMC will be composed of three to five members with experience and expertise in methods, statistics and pediatric critical care collectively. None of the members will be on the steering committee or otherwise involved in the trial to maintain their independence. The primary purpose of the DSMC is to ensure the safety of the children enrolled in the trial. The DSMC will also ensure the credibility of the trial and the validity of its results. The committee will meet and review the available data after 25 and 50% of the patients have been enrolled. Additional meetings may be held at the discretion of the Chair of the DSMC. The committee will receive SAE reports as they occur. All data will be presented to the DSMC tabulated by intervention group but the members will remain blinded to the actual group assignment. The committee will review serious adverse events and centre performance (enrollment, data quality and protocol adherence) and any pertinent external data such as newly published studies or other potentially relevant safety information. The committee will be advisory to the Steering Committee, making any recommendations regarding continuing or suspending the trial enrolment, or modifying trial protocol and procedures. They may recommend early termination of the trial if there are severe adverse events associated with the trial intervention, but no formal stopping rules will be used: this decision will be based on clinical judgment of the DSMC. The DSMC will keep all trial data, committee deliberations, and meeting minutes confidential until the end of the trial.

### Analysis and reporting

All analyses will be performed without knowledge of group assignment and using an intention-to-treat principle. There will be no interim analyses for this pilot trial. For the feasibility outcomes we will report the proportions of children meeting each success criterion and the associated 95% confidence intervals. If, after the completion of the pilot trial, the study Steering Committee determines that there are no important changes to the inclusion and exclusion criteria, the results will not be unblinded for the clinical outcomes of the pilot trial. Instead, we will report the feasibility outcomes, present the clinical outcomes as a single cohort, and consider the pilot trial to be an internal pilot, meaning that we will include the pilot trial patients in the larger RCT. If the Steering Committee judges that a large trial is not feasible or if including the pilot trial patients in the larger RCT is inappropriate, the clinical outcomes will be reported by group so that the trial can be included in future meta-analyses. We will use the SPIRIT and CONSORT guidelines for reporting [23, 24].

## Discussion

Large RCTs are uncommon in pediatric critical care; only 10 trials randomizing more than 500 children have been published [13]. In addition, one-third of published trials are stopped early, most frequently for feasibility problems or futility [13]. Clearly a pilot trial is needed before undertaking such a trial in this relatively small, highly acute and heterogeneous population. Simply conducting a pilot trial will not guarantee the successful completion of a large trial; only 13% of pilot trials in pediatric critical care led to larger trials [25]. This pilot trial evaluates the important threats to the feasibility of a large RCT: screening, accrual, enrollment, and protocol adherence - with specific criteria for considering whether a trial is feasible. The number of participating PICUs in this study will help to ensure that the results are generalizable beyond a few highly-motivated centres.

This pilot trial may suggest that a larger trial is not feasible. It is more likely that this carefully designed pilot trial will ensure that the larger trial we undertake is successful. Within the Canadian Critical Care Trials Group, this programmatic approach has led to large, rigorous and practice-changing trials [26–29]. If the pilot trial leads to a conclusion that a larger trial is feasible, we will conduct a large multicentre trial focusing on clinical outcomes. We propose using the same inclusion and exclusion criteria, and intervention, but these may be modified based on the results of the pilot trial. The objective of the large trial is to determine if a strategy of withholding stress ulcer prophylaxis in critically ill children is not inferior to a strategy of routine stress ulcer prophylaxis. We hypothesize that withholding stress ulcer prophylaxis will not result in an unacceptable increase in upper GI bleeding. An important secondary hypothesis is that there will be fewer nosocomial infections (VAP and CDAD) in the group who do not receive prophylaxis. We will likely use a non-inferiority design because stress ulcer prophylaxis is already commonly used and we wish to test if withholding this conventional treatment results in an important increase in bleeding events. If the increase is small, it may be balanced by avoiding the adverse events and costs associated with prophylaxis which is almost universally administered to critically ill children today.

## Abbreviations

CCCTG: Canadian Critical Care Trials Group
CDAD: Clostridium difficile-associated diarrhea
CI: Confidence interval
DSMC: Data safety monitoring committee
GI: Gastrointestinal
H_2_RA: Histamine-2 receptor antagonist
PICU: Pediatric intensive care unit
PPI: Proton-pump inhibitor
RCT: Randomized controlled trial
REB: Research Ethics Board
RR: Relative risk
VAP: Ventilator associated pneumonia
