# Erratum to: Pediatric intensive care stress ulcer prevention (PIC-UP): a protocol for a pilot randomized trial

**DOI:** 10.1186/s40814-017-0173-4

**Published:** 2017-08-18

**Authors:** Mark Duffett, Karen Choong, Jennifer Foster, Elaine Gilfoyle, Jacques Lacroix, Nikhil Pai, Lehana Thabane, Deborah J Cook, for The Canadian Critical Care Trials Group

**Affiliations:** 1 Departments of Pediatrics and Clinical Epidemiology and Biostatistics, McMaster University, Hamilton, ON Canada; 2 Department of Pediatrics, Dalhousie University, Halifax, NS Canada; 3 Department of Paediatrics, University of Calgary, Calgary, AB Canada; 4 Department of Pediatrics, Université de Montréal, Montreal, Quebec Canada; 5 Department of Pediatrics, McMaster University, Hamilton, ON Canada; 6 Department of Clinical Epidemiology and Biostatistics, McMaster University, Hamilton, ON Canada

## Erratum

Following publication of the original article [1], the authors identified an error in the list of contributing authors. “Deborah J Cook and for The Canadian Critical Care Trials Group” should read “Deborah J Cook for The Canadian Critical Care Trials Group”.

The original article has been corrected.
